# KDP Aqueous Solution-in-Oil Microemulsion for Ultra-Precision Chemical-Mechanical Polishing of KDP Crystal

**DOI:** 10.3390/ma10030271

**Published:** 2017-03-09

**Authors:** Hui Dong, Lili Wang, Wei Gao, Xiaoyuan Li, Chao Wang, Fang Ji, Jinlong Pan, Baorui Wang

**Affiliations:** Institute of Machinery Manufacturing Technology, China Academy of Engineering of Physics (CAEP), Mianyang 621900, China; xinqindonghui@163.com (H.D.); beihangwanglili47@126.com (L.W.); gw0816@foxmail.com (W.G.); lxy20056482@126.com (X.L.); wangchaohit@126.com (C.W.); jifang2004@sina.com (F.J.)

**Keywords:** KDP crystal, chemical-mechanical polishing, water-in-oil microemulsion

## Abstract

A novel functional KH_2_PO_4_ (KDP) aqueous solution-in-oil (KDP aq/O) microemulsion system for KDP crystal ultra-precision chemical-mechanical polishing (CMP) was prepared. The system, which consisted of decanol, Triton X-100, and KH_2_PO_4_ aqueous solution, was available at room temperature. The functional KDP aq/O microemulsion system was systematically studied and applied as polishing solution to KDP CMP technology. In this study, a controlled deliquescent mechanism was proposed for KDP polishing with the KDP aq/O microemulsion. KDP aqueous solution, the chemical etchant in the polishing process, was caged into the micelles in the microemulsion, leading to a limitation of the reaction between the KDP crystal and KDP aqueous solution only if the microemulsion was deformed under the effect of the external force. Based on the interface reaction dynamics, KDP aqueous solutions with different concentrations (*c*_KDP_) were applied to replace water in the traditional water-in-oil (W/O) microemulsion. The practicability of the controlled deliquescent mechanism was proved by the decreasing material removal rate (MRR) with the increasing of the *c*_KDP_. As a result, the corrosion pits on the KDP surface were avoided to some degree. Moreover, the roughnesses of KDP with KDP aq/O microemulsion (*c*_KDP_ was changed from 10 mM to 100 mM) as polishing solutions were smaller than that with the W/O microemulsion. The smallest surface root-mean-square roughness of 1.5 nm was obtained at a 30 mmol/L KDP aq solution, because of the most appropriate deliquescent rate and MRR.

## 1. Introduction

Potassium dihydrogen phosphate (KDP) crystal is an excellent non-linear optic material, which plays a significant role in high-power-density solid-state lasers for inertial confinement fusion (ICF) [1]. High surface quality is important for KDP crystals implemented in these high-power lasers. However, owing to its soft-crisp texture, easy deliquescence, sensitivity to temperature changes and anisotropy, the KDP crystal is a kind of difficult machining optical component, which makes it difficult to get a super-smooth and super-clean surface through traditional methods [2]. At present, single-point diamond turning (SPDT) and magnetorheological finishing (MRF) are widely applied in KDP ultra-precision matching [3,4,5,6,7,8]. Ultra-precision grinding was also studied and resulted in high-quality KDP crystals with a surface root-mean-square (RMS) roughness of 0.553 nm [9]. However, these methods have inherent disadvantages, such as micro-scale ripples from SPDT processing, abrasive embedment on the finished surface and surface fogging after MRF processing.

Chemical mechanical polishing (CMP) is supposed to be a promising technology to avoid the above disadvantages [10,11] and was introduced to the KDP matching area to achieve a super-smooth and super-clean KDP surface. Typically, a CMP slurry is an aqueous dispersion containing abrasive particles, an activating agent, a passivating agent, and a surfactant. It is extremely difficult to polish KDP crystals via the traditional CMP slurry, because KDP crystals are easily damaged by water and abrasive particles. In the past 10 years, appropriative non-abrasive CMP solutions for KDP crystals have been researched based on the micro-deliquescence of KDP [12,13]. More recently, a new abrasive-free system based on a water-in-oil (W/O) microemulsion was developed for a polishing solution of KDP CMP [14]. In this unique polishing system, the reaction between the KDP and water was controlled by caging water into micelles. During the polishing process, the frictional action between the crystal surface and pad led to the release of water, which dissolved the KDP from the crystal surface. They obtained a scratch-free polished KDP surface with a surface RMS roughness of 1.7 nm [15].

Although the polishing mechanism of KDP CMP gained a great breakthrough with traditional W/O microemulsion, drawbacks remained. Macroscopically, the deliquescence reaction between the KDP crystal and water was limited by caging water droplets into micelles. However, at the microcosmic level, the interface reaction was essentially invariant. Actually, deliquescence in every tiny area was severe and wild, which resulted in corrosion pits at the KDP surface. Therefore, it was necessary to modulate the interface reaction dynamics at the molecular level. In this study, a novel abrasive-free polishing solution system based on a KDP aqueous solution-in-oil microemulsion (KDP aq/O) was prepared and its potential application in KDP ultra-precision CMP was investigated systematically. The controlled deliquescent mechanism was proposed for KDP polishing with the KDP aq/O microemulsion. In the KDP polishing process, the KDP aqueous solution (KDP aq), as the chemical etchant, was caged into the micelles in the microemulsion, so that the reaction between the KDP crystal and KDP aq was limited when the microemulsion was deformed under the effect of external force. Comprehensive optimization of the chemical-mechanical polishing experiments was carried out according to the controlled deliquescent principle.

## 2. Experimental Section

### 2.1. Partial Phase Diagram

In order to determine the chemical composition of microemulsion, the partial ternary phase diagram was constructed at room temperature, which was consistent with the result in published literature [14]. In decanol/Triton X-100/KDP aq system, *c*_KDP_ was from 10 mM to 100 mM. For comparison, ternary system decanol/Triton X-100/water was also researched. A series of samples with different weight ratios of decanol (the continuous phase) to Triton X-100 (the surfactant) were prepared. Afterwards, the dispersed phase (KDP aq with certain concentration or pure water) was added drop wisely to the above mixtures under firmly stirring. KDP aq/O or W/O region in the phase diagram was determined by two points: One was the change of mixture appearance from clear to cloudy and the other was from cloudy to clear. The performance on the process of KDP CMP was then discussed. All experiments were carried out at 20 °C and 40% relative humidity (RH).

### 2.2. Sample Preparation

All sample preparation and polishing process was carried out at 20 °C, 40% RH. Polishing solution played an important role in CMP processing and determined the quality of polished KDP surface. In order to investigate the functional effect of KDP aq in microemulsion, the weight ratio of the continuous phase (decanol), the surfactant (Triton X-100) and the dispersed phase (KDP aq with certain concentration or pure water) was maintained at 50:40:10, and *c*_KDP_ was set as a variable.

### 2.3. Materials Characterizations of Microemulsion

Viscosity was measured with capillary viscometers. The micellar sizes in dispersion was measured on a non-invasive back scatter system (ALV-GmbH, Langen, Germany), a particle sizer based on dynamic light scattering (DLS) technique. All experiments were carried out at 20 °C.

### 2.4. Chemical-Mechanical Polishing of KDP Crystal

**Figure 1:**
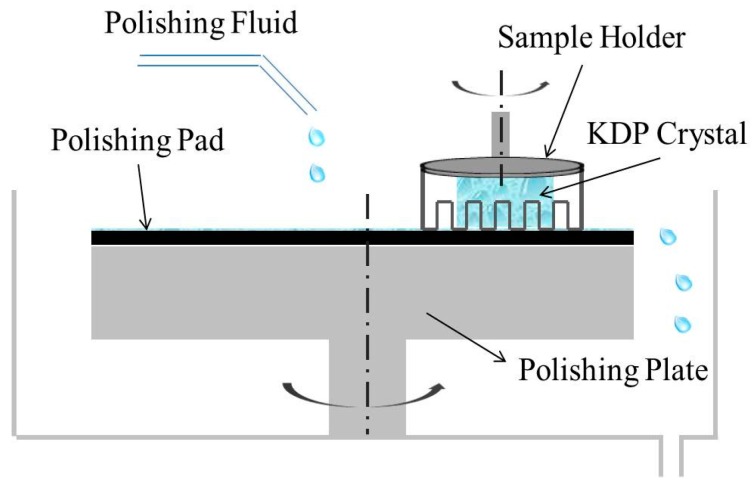
Schematic diagram of chemical mechanical polishing technology for KDP crystals.

KDP crystals (35 × 35 × 10 mm^3^) used in the experiments were provided by Shandong University. Before CMP, each KDP crystal was preprocessed by SPDT. Figure 1 showed the schematic diagram of KDP CMP. Polishing plate (Φ 230 mm) was actively driven by motor carrier. KDP crystal was stuck onto a stainless steel holder using wax, and then mounted on polishing cushion, which was rotated with the same direction as polishing plate to remove material uniformly. Polishing pressure was changed by adjusting the load capacity on KDP crystal. Under the centrifugal force, polishing solution (KDP aq/O or W/O) was distributed uniformly on polishing pad and formed a film between KDP crystal and pad. Under the effect of pressure and friction force, KDP aq was released from microemulsion and reached to KDP surface to achieve polishing. CMP experiments using KDP aq/O microemulsion as polishing solution were labeled as CMP-KDP aq/O, while CMP experiments using W/O microemulsion as CMP-W/O. Typically, polishing time was 15 min, pressure was 5 kPa, and the platen speed was 60 rpm. After polishing, KDP crystal was washed by isopropanol, acetic acid, and cyclohexane, respectively.

The surface quality was the key parameter for most optical systems. An optical microscopy by Nikon Epiphot 300 (Nikon, Tokyo, Japan) was employed to examine the topography and characteristics of the separated surface. A Taylor Hobson CCI lite with a 20× objective and full resolution was used to examine the surface roughness and spatial frequency to analyze the surface texture. Material removal rate (MRR) was tested by height-measuring equipment. All experiments were carried out at 20 °C and 40% RH.

## 3. Results and Discussion

### 3.1. Phase Diagram and Microemulsion Selection

**Figure 2:**
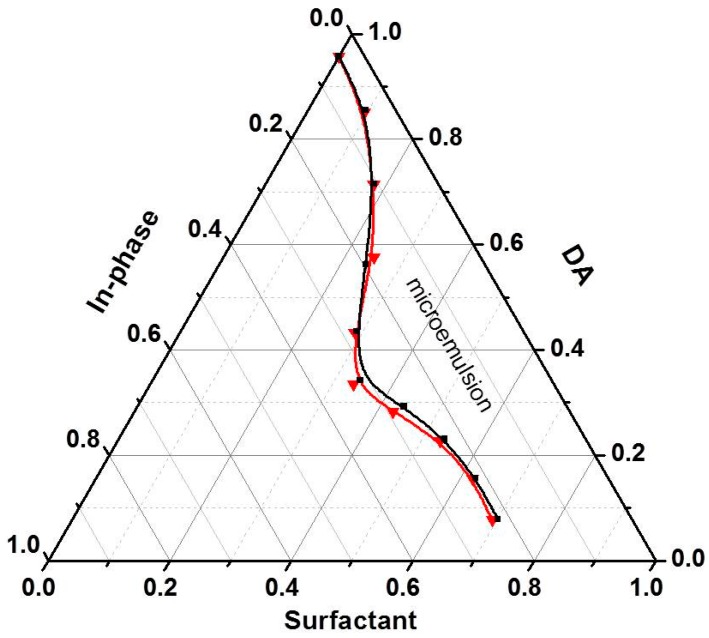
Partial phase diagram for systems of decanol/Triton X-100/water (black line) and decanol/Triton X-100/KDP aq (red line).

**Figure 3:**
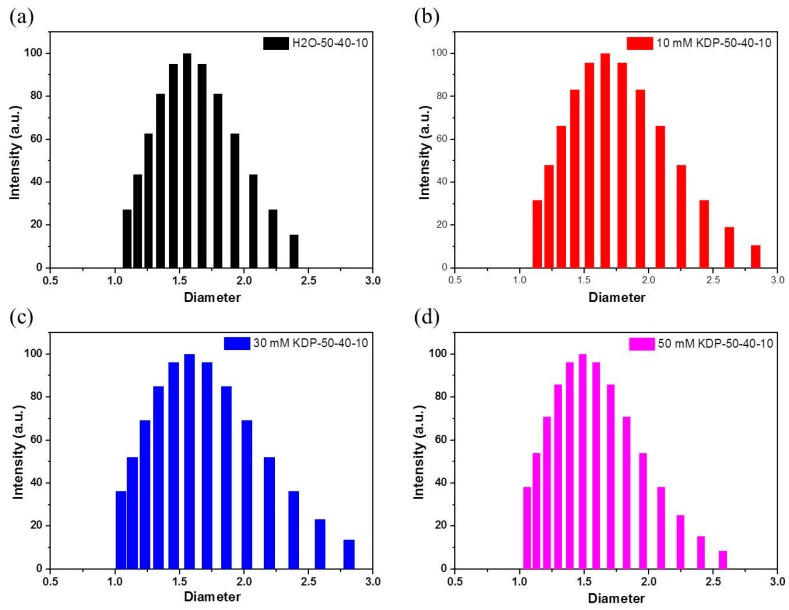
Size distribution of micelles in traditional W/O microemulsion (**a**) and functional KDP aq/O microemulsion with different *c*_KDP_ (**b**–**d**).

The polishing solution played an important role in CMP processing and determined the quality of the polished KDP surface. The partial phase diagram for the decanol/Triton X-100/KDP aq system (Figure 2) was constructed at room temperature, which was similar to that of the decanol/Triton X-100/water ternary system. A suitable W/O microemulsion solution for KDP CMP was required to obtain the advantages of appropriate viscosity, good flow ability, low volatility and proper number and size of micelles. So all the CMP solution was prepared with the same stoichiometry (50:40:10). In order to study the controlled deliquescent mechanism, functional microemulsions with different *c*_KDP_ were prepared. Figure 3 shows the size distribution of micelles in the traditional W/O microemulsion (Figure 3a) and the KDP aq/O microemulsion (Figure 3b–d). It was found that the size distribution of the micelles remained approximately unchanged with the increase of *c*_KDP_.

### 3.2. CMP Based on Controlled Deliquescent Mechanism

**Figure 4:**
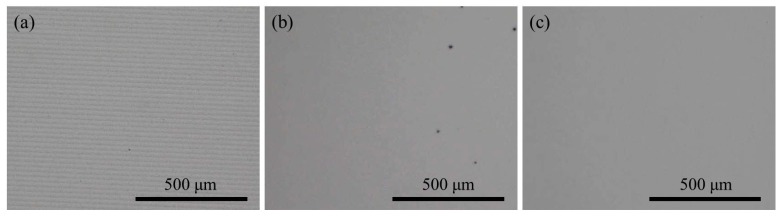
Surfacial topographies of KDP samples: (**a**) KDP sample after SPDT; (**b**) KDP sample after CMP with traditional W/O microemulsion; and (**c**) KDP sample after CMP with KDP aq/O microemulsion.

Chemical mechanical polishing experiments were carried out with KDP aq/O and W/O, respectively. Figure 4 shows the surface topographies of KDP samples. After the experiments of SPDT, micro-scale ripples formed on the surface of the KDP crystal (Figure 4a). As shown Figure 4b,c, ripples from the SPDT were wiped off after CMP with W/O or KDP aq/O. In the case of CMP-W/O, water molecules were released from the W/O microemulsion and reached the KDP surface to achieve polishing. Actually, the deliquescence in every tiny area was severe and wild, so corrosion pits were formed on the KDP surface. By using the KDP aqueous solution instead of water, corrosion pits could not be detected in the case of the CMP-KDP aq/O.

**Figure 5:**
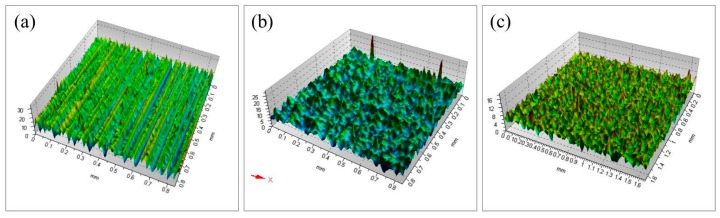
Surface microstructures of different KDP samples: (**a**) KDP after SPDT; (**b**) KDP after CMP with traditional W/O microemulsion; and (**c**) KDP after CMP with KDP aq/O microemulsion (*c*_KDP_ was 30 mM).

The microstructures are shown in Figure 5. The results showed that the surface of the KDP crystal polished by SPDT became flatter after the processes of CMP-KDP aq/O and CMP-W/O. In addition, the smoothest surface was obtained by CMP-KDP aq/O. The surface RMS roughness was reduced from 4.6 nm by SPDT to 3.1 nm after polishing with W/O or 1.5 nm after polishing with KDP aq/O (as shown in Table 1). The result of 3.1 nm for the W/O microemulsion was different from that of Reference [14], due to different polishers, different sample sizes, and different polishing parameters (press or movement trace) [16]. It showed that KDP aq/O in a decanol/Triton X-100/KDP aq. system is a promising CMP solution for KDP crystals, which reduced the surface roughness by wiping off ripples from the SPDT. Meanwhile, KDP aq/O prevented the formation of corrosion pits.

### 3.3. Research of Material Removal Rate (MRR)

In order to understand the influence that the controlled deliquescent mechanism attributed to the improvement of the surface RMS roughness, we researched the material removal rate by using KDP aq/O with different *c*_KDP_. It was known that the material removal rate of the KDP crystal is determined by mechanical grinding and deliquescent action. In this work, the deliquescent action was solely variable, while the mechanical grinding was invariable due to stationary polishing condition and environment. Therefore, the value of MRR depended on the deliquescent rate of the KDP crystal. In the CMP processes, W/O or KDP aq/O microemulsion was deformed under the effect of pressure and friction force. Water or KDP aq reached the surface of the KDP crystal and gave rise to the deliquescent reaction (equation is shown as follows). According to the reaction kinetics, the MRR of the KDP crystal showed an inverse correlation to *c*_KDP_.

$$\mathrm{KDP}\;\mathrm{Crystal}(s)\overset{\mathrm{KDP}\;\mathrm{aq}\;\mathrm{from}\;\mathrm{deformed}\;\mathrm{KDP}\;\mathrm{aq}/O}{\underset{\mathrm{or}\;\mathrm{water}\;\mathrm{from}\;\mathrm{deformed}\;W/O}{\to }}\mathrm{KDP}\;\mathrm{aq}(l)$$

**Figure 6:**
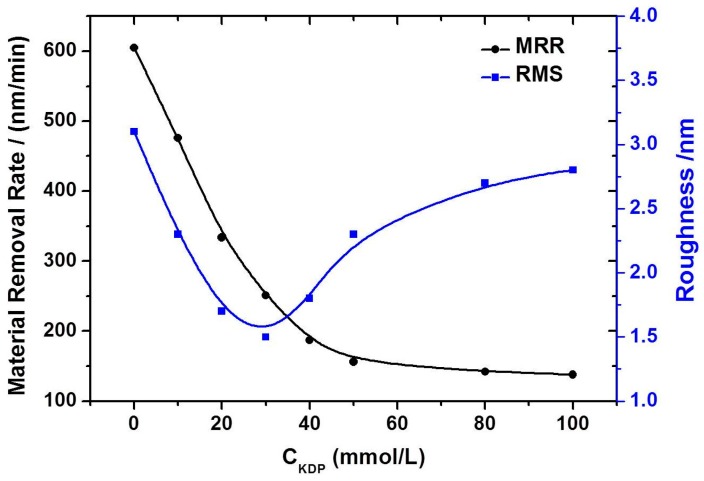
Material removal rate and roughness comparison with different *c*_KDP_ (from 0 mM to 100 mM).

For in-depth insight into the controlled deliquescent mechanism, the MRR of KDP aq/O with different *c*_KDP_ was researched. Figure 6 shows the experimental results of the MRR and the roughness comparison with different *c*_KDP_. The corresponding parameters are listed in Table 2. It was found that *c*_KDP_ had a great influence on the MRR and surface RMS roughness. The MRR decreased when the *c*_KDP_ increased from 0 to 100 mM, which was consistent with the kinetics of the deliquescent reaction. Moreover, the roughnesses of the KDP crystal with KDP aq/O (*c*_KDP_ was changed from 10 mM to 100 mM) as the polishing solution were smaller than that with the W/O microemulsion. The smallest surface RMS roughness of 1.5 nm was obtained with a 30 mmol/L KDP aq solution, with an appropriate MRR of 251 nm/min. However, when the *c*_KDP_ was greater than 30 mM, the effect of the deliquescent action became weaker than that of the mechanical grinding, which led to the increase of the surface RMS roughness.

## 4. Conclusions

In this paper, a functional KDP/O microemulsion was systematically researched and applied as a polishing solution for KDP chemical-mechanical polishing. The KDP aq/O microemulsion system was homogeneous and transparent on a macroscopic scale and kept a similar phase diagram as the traditional W/O microemulsion. Compared with the traditional W/O microemulsion, the KDP aq/O microemulsion reduced the MRR of CMP observably, which was beneficial for avoiding corrosion pits and improving the surface quality of the surface RMS roughness. We studied the effect of *c*_KDP_ on the MRR and surface RMS roughness. It was demonstrated that MRR decreased when the *c*_KDP_ increased from 0 mM to 100 mM while a highly smooth surface was obtained when the *c*_KDP_ was 30 mM with a corresponding MRR of 251 nm/min. Comprehensive optimization of polishing experiments should be carried out to explore the principle of ultra-precision polishing and to obtain the best technological parameters (pressure, revolving speed, and polishing time) for chemical-mechanical polishing.

## Figures and Tables

**Table 1:** RMS roughness for KDP crystal after experiments of SPDT, CMP-1 and CMP-2 (polishing condition: temperature 20 °C, humidity 40% RH, pressure 5 kPa).

Experiments	Surface Quality	RMS Roughness/nm
SPDT	Micro-scale ripples	4.6
CMP-W/O	Corrosion pits	3.1
CMP-KDP aq/O	Smooth	1.5

**Table 2:** Parameters for different microemulsions and their corresponding MRRs for the KDP crystal (polishing condition: temperature 20 °C, humidity 40% RH, pressure 5 kPa).

CMP Solutions	*c*_KDP_/mM	MRR/(nm/min)	RMS/nm
1	0	605	3.1
2	10	476	2.3
3	20	334	1.7
4	30	251	1.5
5	40	187	1.8
6	50	156	2.3
7	80	142	2.7
8	100	138	2.8

## Supplementary Materials

The following are available online at www.mdpi.com/1996-1944/10/3/271/s1. Figure S1: Outline of crossing section for different KDP samples: (a) KDP after SPDT; (b) KDP after CMP with traditional W/O microemulsion; and (c) KDP after CMP with functional KDP aq/O microemulsion (*c*_KDP_ was 30 mM).
