# Supplementary material for: KDP Aqueous Solution-in-Oil Microemulsion for Ultra-Precision Chemical-Mechanical Polishing of KDP Crystal
Source: Materials (Basel). 2017 Mar 9;10(3):271. doi: 10.3390/ma10030271 (PMC5503384; doi:10.3390/ma10030271)
Supplement: Supplementary file 1 [file materials-10-00271-s001.pdf]

Supplementary Materials: KDP Aqueous Solution-in-Oil Microemulsion for Ultra-Precision Chemical-Mechanical Polishing of KDP Crystal

Hui Dong, Lili Wang, Wei Gao, Xiaoyuan Li, Chao Wang, Fang Ji, Jinlong Pan and Baorui Wang

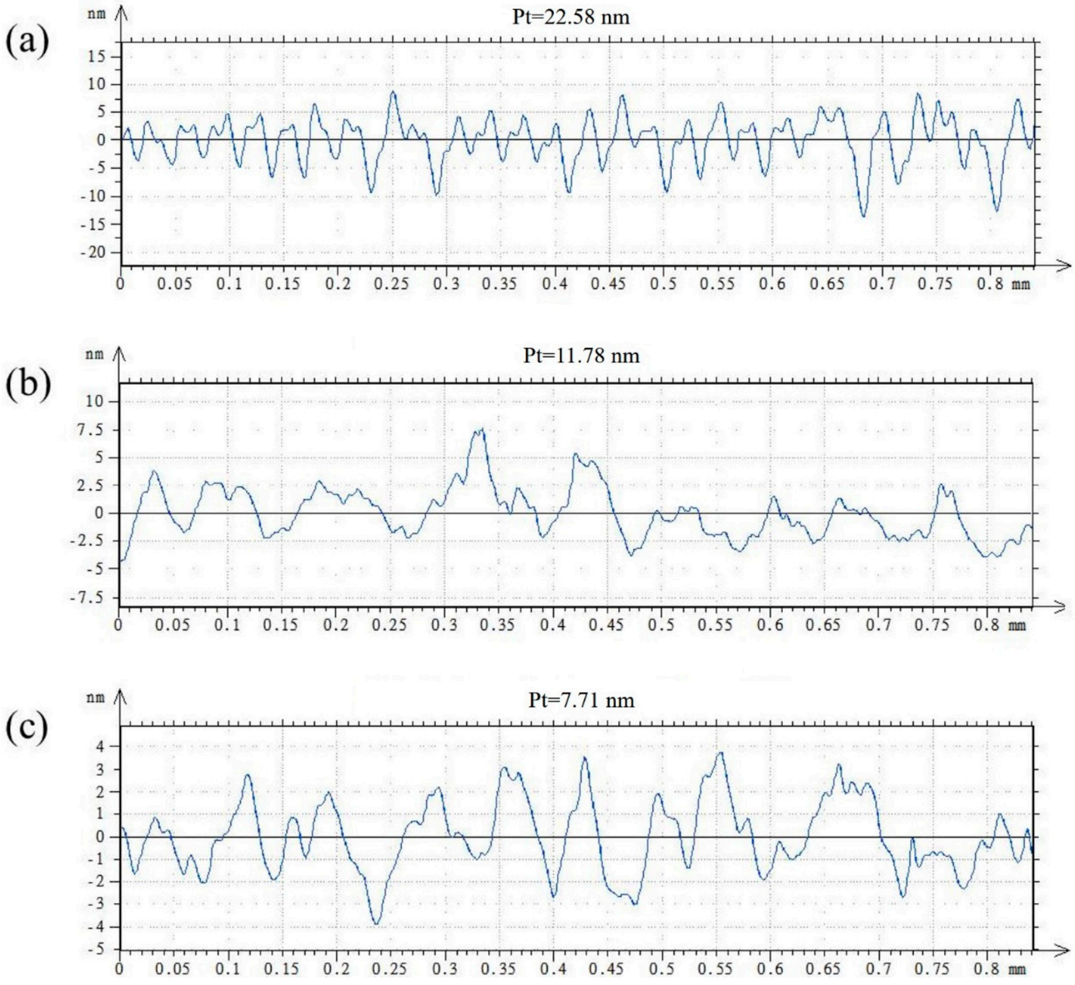

Figure S1. Outline of crossing section for different KDP samples: (**a**) KDP after SPDT; (**b**) KDP after CMP with traditional W/O microemulsion; and (**c**) KDP after CMP with functional KDP aq/O microemulsion (c_{KDP} was 30 mM).
